# Supplementary material for: Predictive Roles of Basal Metabolic Rate and Body Water Distribution in Sarcopenia and Sarcopenic Obesity: The link to Carbohydrates
Source: Nutrients. 2022 Sep 21;14(19):3911. doi: 10.3390/nu14193911 (PMC9571591; doi:10.3390/nu14193911)
Supplement: Supplementary file 1 [file nutrients-14-03911-s001.zip › nutrients-1890732-supplementary.pdf]

Supplementary File

Supplementary Table S1. Demographic and clinical characteristics

Supplementary Table S2. The consumption of macronutrients and micronutrients between different population

Supplementary Table S3. Association between BMR, body composition, dietary components and SMI

Supplementary Table S4. Determinate important factors for predicting sarcopenia and sarcopenic obesity

Supplementary Table S5. Predictive role of BMR and ECW/ICW

Supplementary Table S6. Predictive role of BMR and ECW/TBW

Supplementary Table S7. Correlations among SMI, BMR, carbohydrates intake and body water distribution

Supplementary Table S8. Moderated mediation analysis for carbohydrates, BMR, SMI and bogy water distribution

Supplementary Table S1. Demographic and clinical characteristics

Characteristics	Total	Healthy	Sarcopenia	Sarcopenic obesity	Obesity	*P*
TOTAL	402	118	20	12	252	
Sex, n(%)						
Men	240	78(66.1)	12 (60.0)	6(50.0)	144(57.1)	0.367
Women	162	40(33.9)	8 (40.0)	6(50.0)	108(42.9)	
Age groups, n(%)						
50-64years	101	34(28.8)	1(5.0)	0(0.0)	66(26.2)	<0.001***
65-79years	264	75(63.6)	11(55.0)	9(75.0)	169(67.1)	
80 years and older	37	9(7.6) [a]	8(40.0) [a, b]	3(25.0)	17(6.7) [b]	
BMI, n(%)						
＜18.5	20	13(11.0) [a]	7(35.0) [a]	0(0.0)	0(0.0)	<0.001***
18.5-23.9	173	90(76.3) [a]	13(65.0) [b]	8(66.7) [c]	62(24.6) [a, b, c]	
24-27.9	168	15(12.7) [a]	0(0.0)	3(25.0)	150(59.5) [a]	
≥28	41	0(0.0)	0(0.0)	1(8.3)	40(15.9)	
Blood biomarkers						
Blood glucose	5050(5.00,6.20)	5.21(1.80,5.83) [a, b]	5.21(4.80,5.83) [c, d]	5.79(5.52,6.33) [a, c]	5.60(5.10,6.40) [b, d]	0.001**
Hb	139.00(129.00,151.00)	141.59±16.15	129.15±8.90	141.17±23.21	140.00(129.00,151.00)	0.06
TG	1.39(1.09,1.98)	1.18(0.96,1.50) [a]	1.13±0.37 [b]	1.67(1.01,1.87)	1.57(1.16,2.28) [a, b]	<0.001***
TC	5.46(4.73,6.26)	5.37±1.29	5.38±1.16	5.49±1.74	5.49±1.34	0.854

Continuous variables are shown as mean ± standard deviation for variables with normal distribution, median (interquartile range [IQR]) for nonnormal distributions. Categorical variables are shown as n (%).

BMI, Body mass index; Hb, Hemoglobin; TG, Triglyceride; TC, Serum total cholesterol.

[a, b, c] declared $P < 0.05$ between two means with the same upper letter in the same line.

** $P < 0.01$, *** $P < 0.001$

Supplementary Table S2. The consumption of macronutrients and micronutrients between different population

	Healthy			Sarcopenia			Sarcopenic obesity			Obesity			*P*
	< EAR	> EAR	> RNI	< EAR	> EAR	> RNI	< EAR	> EAR	> RNI	< EAR	> EAR	> RNI	
Macronutrients													
Carbohydrates (g/d)	3(2.5)	23(19.5)	92(78.0)	1(5.0)	4(20.0)	15(75)	0(0.0)	2(16.7)	10(83.3)	2(0.8)	39(15.5)	211(83.7)	0.386
Total proteins (g/d)	67(56.8)	13(11.0)	38(32.2)	9(45.0)	1(5.0)	10(50.0)	8(66.7)	2(16.7)	2(16.7)	144(57.1)	22(8.7)	86(34.1)	0.557
Micronutrients													
Magnesium (mg/d)	39(33.1)	31(26.3)	48(40.7)	4(20.0)	5(25.0)	11(55.0)	4(33.3)	3(25.0)	5(41.7)	67(26.6)	74(29.4)	111(44.0)	0.812
Phosphorus (mg/d)	5(4.2)	15(12.7)	98(83.1)	1(5.0)	1(5.0)	18(90.0)	0(0.0)	2(16.7)	10(83.3)	2(0.8)	28(11.1)	222(88.1)	0.192
Ferrum (mg/d)	0(0.0)	0(0.0)	118(100.0)	0(0.0)	0(0.0)	20(100.0)	0(0.0)	0(0.0)	12(100.0)	0(0.0)	0(0.0)	252(100.0)	—
Calcium (mg/d)	100(84.7)	7(5.9)	11(9.3)	17(85.0)	1(5.0)	2(10.0)	9(75.0)	2(16.7)	1(8.3)	195(77.7)	22(8.8)	34(13.5)	0.640
Vitamin A (μg/d)	53(44.9)	12(10.2)	53(44.9)	8(40.0)	1(5.0)	11(55.0)	7(58.3)	0(0.0)	5(41.7)	128(50.8)	25(9.9)	99(39.3)	0.646
Vitamin B1 (μg/d)	113(95.8)	4(3.4)	1(0.8)	20(100.0)	0(0.0)	0(0.0)	11(91.7)	0(0.0)	1(8.3)	229(90.9)	15(6.0)	8(3.2)	0.399
Vitamin B2(μg/d)	0(0.0)	0(0.0)	118(100.0)	0(0.0)	0(0.0)	20(100.0)	0(0.0)	0(0.0)	12(100.0)	0(0.0)	0(0.0)	252(100.0)	—
Vitamin B3(μg/d)	39(33.1)	38(32.2)	41(34.7)	5(25.0)	7(35.0)	8(40.0)	5(41.7)	3(25.0)	4(33.3)	82(32.5)	62(24.6)	108(42.9)	0.647
Vitamin C (mg/d)	34(29.1)	5(4.3)	78(66.7)	3(15.0)	2(10.0)	15(75.0)	3(25.0)	1(8.3)	8(66.7)	66(26.2)	12(4.8)	174(69.0)	0.667
	< EER		> EER	< EER		> EER	< EER		> EER	< EER		> EER	*P*
Total energy (kcal/d)	109(92.4)		9(7.6)	18(90.0)		2(10.0)	11(91.7)		1(8.3)	211(83.7)		41(16.3)	0.114
	⩽AMDR		> AMDR	⩽AMDR		> AMDR	⩽AMDR		> AMDR	⩽AMDR		> AMDR	*P*
Carbohydrate density (%/E)	117(99.2)		1(0.8) [a, b]	20(100.0)		0(0.0)	11(91.7)		1(8.3) [a]	236(93.7)		16(6.3) [b]	0.046*
Lipids density (%/E)	0(0.0)		118(100.0)	0(0.0)		20(100.0)	0(0.0)		12(100.0)	5(2.0)		247(98.0)	0.461
	< AI		> AI	< AI		> AI	< AI		> AI	< AI		> AI	*P*

Manganese (mg/d)	0(0.0)	118(100.0)	0(0.0)	20(100.0)	0(0.0)	12(100.0)	3(1.2)	228(98.8)	0.653
Potassium (mg/d)	84(71.2)	34(28.8)	9(45.0)	11(55.0)	9(75.0)	3(25.0)	157(62.3)	95(37.7)	0.079

Categorical variables are shown as n (%).

[a, b] declared $P < 0.05$ between two means with the same upper letter in the same line.

[*] $P < 0.05$

Supplementary Table S3. Association between BMR, body composition, dietary components and SMI

Factor included	MODEL 3			MODEL 4		
	Unstandardized	Standardized	*P*	Unstandardized	Standardized	*P*
	B(95%CI)	β		B(95%CI)	β	
Sex	-0.283	-0.139	<0.001***	-0.283	-0.139	<0.001***
Age	-0.004	-0.025	0.183	-0.004	-0.025	0.182
BMR	0.006	1.041	<0.001***	0.006	1.041	<0.001***
ECW/ICW	-3.597	-0.070	<0.001***			
ECW/TBW				-9.579	-0.069	<0.001***
Percentage Body Fat (%)	0.038	0.287	<0.001***	0.038	0.287	<0.001***
WHR	-4.256	-0.255	<0.001***	-4.252	-0.255	<0.001***
Blood glucose	0.001	0.002	0.897	0.001	0.002	0.898
Hb	-0.002	-0.030	0.089	-0.002	-0.030	0.091
TG	0.015	0.018	0.280	0.015	0.018	0.281
Total energy (kcal/d)	<0.001	-0.125	0.025*	<0.001	-0.125	0.025*
Carbohydrates density (%/E)	-0.034	-0.271	0.042*	-0.034	-0.271	0.042*
Protein density (%E)	-0.030	-0.087	0.066	-0.030	-0.087	0.066
Lipids density (%/E)	-0.034	-0.242	0.039*	-0.034	-0.241	0.039*
Dietary fiber (g/d)	0.004	0.024	0.398	0.004	0.024	0.400

WHR, Waist hip ratio; TBW, Total body water; ICW, Intracellular water; ECW, Extracellular water; BMR, Basal metabolic rate; SMI, Appendicular skeletal muscle index; Hb, Hemoglobin; TG, Triglyceride; TC, Serum total cholesterol.

*P<0.05, *** P<0.001

Supplementary Table S4. Determinate important factors for predicting sarcopenia and sarcopenic obesity

	Sensitivity	Specificity	Cut-off value
BMR			
Healthy vs. Sarcopenia	0.900	0.568	1316.2570
Healthy vs. Sarcopenic obesity	0.917	0.661	1271.2500
Sarcopenia vs. Sarcopenic obesity	0.833	0.450	1256.6730
BMR/BMI			
Healthy vs. Sarcopenia	0.250	0.881	70.4416
Healthy vs. Sarcopenic obesity	0.917	0.746	56.8966
Sarcopenia vs. Sarcopenic obesity	0.917	0.750	57.2843
BMR/BSA			
Healthy vs. Sarcopenia	0.900	0.525	832.2929
Healthy vs. Sarcopenic obesity	0.917	0.720	810.7782
Sarcopenia vs. Sarcopenic obesity	0.750	0.800	791.1238
BMR/Height^2			
Healthy vs. Sarcopenia	0.750	0.720	0.0479
Healthy vs. Sarcopenic obesity	0.500	0.695	0.0481
Sarcopenia vs. Sarcopenic obesity	0.833	0.600	0.0474
ECW/ICW			
Healthy vs. Sarcopenia	0.667	0.644	0.6348
Healthy vs. Sarcopenic obesity	0.850	0.720	0.6370
Sarcopenia vs. Sarcopenic obesity	0.583	0.850	0.6373
ECW/TBW			
Healthy vs. Sarcopenia	0.850	0.720	0.3891
Healthy vs. Sarcopenic obesity	0.667	0.644	0.3883
Sarcopenia vs. Sarcopenic obesity	0.583	0.850	0.3892

BMI, Body mass index; BMR, Basal metabolic rate; BSA, Body surface area; TBW, Total body water; ICW, Intracellular water; ECW, Extracellular water.

Supplementary Table S5. Predictive role of BMR and ECW/ICW

	MODEL 3 BMR		MODEL 3 BMR/BMI		MODEL 3 BMR/BSA		MODEL 3 BMR/ Height2	
	OR(95%CI)	*P*	OR(95%CI)	*P*	OR(95%CI)	*P*	OR(95%CI)	*P*
**Healthy vs. Sarcopenia**								
BMR	0.042 (0.007,0.249)	0.001**						
BMR/BMI			2.621 (0.619,11.088)	0.190				
BMR/BSA					0.078 (0.014,0.428)	0.003**		
BMR/Height^2							0.212 (0.061,0.737)	0.015*
ECW/ICW	7.251 (1.704,30.854)	0.007**	5.573 (1.443,21.526)	0.013*	4.346 (1.077,17.530)	0.039*	4.148 (1.030,16.713)	0.045*
**Healthy vs. Sarcopenic obesity**								
BMR	0.006 (0.000,0.087)	<0.001***						
BMR/BMI			0.002 (0.000,0.053)	<0.001***				
BMR/BSA					—	0.998		
BMR/Height^2							0.766 (0.167,3.524)	0.732
ECW/ICW	1.703 (0.376,7.719)	0.490	2.459 (0.461,13.118)	0.292	1.739 (0.301,10.064)	0.537	1.593 (0.437,5.807)	0.481
**Sarcopenia vs. Sarcopenic obesity**								
BMR	0.217 (0.018,2.601)	0.228						
BMR/BMI			—	0.999				
BMR/BSA					—	0.999		
BMR/Height^2							20.563 (1.304,324.336)	0.032*
ECW/ICW	0.088 (0.011,0.678)	0.020*	0.109 (0.008,1.501)	0.098	0.055 (0.001,0.750)	0.030*	0.097 (0.010,0.935)	0.044*

MODEL3: Model 2 + ECW/ICW

BMI, Body mass index; BMR, Basal metabolic rate; BSA, Body surface area; ICW, Intracellular water; ECW, Extracellular water.

$^{*}P<0.05$, $^{**}P<0.001$, $^{***}P<0.001$

Supplementary Table S6. Predictive role of BMR and ECW/TBW

	MODEL 4 BMR		MODEL 4 BMR/BMI		MODEL 4 BMR/BSA		MODEL 4 BMR/ Height2	
	OR(95%CI)	*P*	OR(95%CI)	*P*	OR(95%CI)	*P*	OR(95%CI)	*P*
**Healthy vs. Sarcopenia**								
BMR	0.046 (0.007,0.280)	0.001**						
BMR/BMI			2.953 (0.655,13.317)	0.159				
BMR/BSA					0.084 (0.015,0.478)	0.005**		
BMR/Height^2							0.259 (0.074,0.914)	0.036*
ECW/TBW	10.062 (2.414,41.942)	0.002**	9.119 (2.359,35.244)	0.001**	6.991 (1.740,28.091)	0.006**	6.582 (1.609,26.914)	0.009**
**Healthy vs. Sarcopenic obesity**								
BMR	0.005 (0.000,0.079)	<0.001***						
BMR/BMI			0.001 (0.000,0.042)	<0.001***				
BMR/BSA					—	0.998		
BMR/Height^2							0.807 (0.173,3.760)	0.784
ECW/TBW	2.923 (0.512,16.683)	0.227	6.336 (0.636,63.152)	0.116	4.748 (0.473,47.633)	0.185	1.968 (0.503,7.696)	0.331
**Sarcopenia vs. Sarcopenic obesity**								
BMR	0.221 (0.020,2.492)	0.222						
BMR/BMI			—	0.998				
BMR/BSA					—	0.998		
BMR/Height^2							15.522 (1.171,205.743)	0.038*
ECW/TBW	0.125 (0.016,0.948)	0.044*	0.171 (0.012,2.358)	0.187	0.076 (0.006,1.001)	0.050	0.206 (0.024,1.786)	0.152

MODEL 4: Model 2 + ECW/TBW

BMI, Body mass index; BMR, Basal metabolic rate; BSA, Body surface area; TBW, Total body water; ECW, Extracellular water.

$^{*}P<0.05$, $^{**}P<0.01$, $^{***}P<0.001$

Supplementary Table S7. Correlations among SMI, BMR, carbohydrates intake and body water distribution

	SMI	BMR	Carbohydrates	ECW/ICW	ECW/TBW
SMI	1				
BMR	0.941***	1			
Carbohydrates	0.222***	0.250***	1		
ECW/ICW	-0.273***	-0.244***	-0.073	1	
ECW/TBW	-0.273***	-0.244***	-0.074	1.000***	1

BMR, Basal metabolic rate; TBW, Total body water; ICW, Intracellular water; ECW, Extracellular water; SMI, Appendicular skeletal muscle index.

*** $P<0.001$

Supplementary Table S8. Moderated mediation analysis for carbohydrates, BMR, SMI and bogy water distribution

	Effect	Boot SE	Boot LLCI	Boot ULCI
ECW/ICW				
Low (0.6154)	0.0036	0.0010	0.0016	0.0055
Moderate (0.6310)	0.0039	0.0011	0.0018	0.0060
High (0.6490)	0.0042	0.0012	0.0019	0.0065
ECW/TBW				
Low (0.3810)	0.0036	0.0010	0.0017	0.0056
Moderate (0.3869)	0.0039	0.0011	0.0018	0.0060
High (0.3936)	0.0042	0.0012	0.0019	0.0065

SE, Standard Error; LLCI, Lower limit confidence interval; ULCI, Upper limit confidence interval; BMR, Basal metabolic rate; TBW, Total body water; ICW, Intracellular water; ECW, Extracellular water; SMI, Appendicular skeletal muscle index
